# The pilocarpine model of temporal lobe epilepsy

**DOI:** 10.1016/j.jneumeth.2008.04.019

**Published:** 2008-07-30

**Authors:** Giulia Curia, Daniela Longo, Giuseppe Biagini, Roland S.G. Jones, Massimo Avoli

**Affiliations:** a Montreal Neurological Institute and Departments of Neurology & Neurosurgery and Physiology, McGill University, Montreal, QC, Canada H3A 2B4; b Dipartimento di Scienze Biomediche, Università di Modena e Reggio Emilia, 41100 Modena, Italy; c Department of Pharmacy and Pharmacology, University of Bath, Bath BA2 7AY, United Kingdom; d Dipartimento di Medicina Sperimentale, Università di Roma “La Sapienza”, 00185 Roma, Italy

**Keywords:** AEDs, antiepileptic drugs, CA, Cornu Ammonis, EEG, electroencephalogram, i.p., intraperitoneal, MRI, magnetic resonance imaging, P, postnatal, s.c., subcutaneous, SE, *status epilepticus*, SRSs, spontaneous recurrent seizures, TLE, temporal lobe epilepsy, Animal models, Entorhinal cortex, Hippocampus, Pilocarpine, Temporal lobe epilepsy

## Abstract

Understanding the pathophysiogenesis of temporal lobe epilepsy (TLE) largely rests on the use of models of *status epilepticus* (SE), as in the case of the pilocarpine model. The main features of TLE are: (i) epileptic foci in the limbic system; (ii) an “initial precipitating injury”; (iii) the so-called “latent period”; and (iv) the presence of hippocampal sclerosis leading to reorganization of neuronal networks. Many of these characteristics can be reproduced in rodents by systemic injection of pilocarpine; in this animal model, SE is followed by a latent period and later by the appearance of spontaneous recurrent seizures (SRSs). These processes are, however, influenced by experimental conditions such as rodent species, strain, gender, age, doses and routes of pilocarpine administration, as well as combinations with other drugs administered before and/or after SE. In the attempt to limit these sources of variability, we evaluated the methodological procedures used by several investigators in the pilocarpine model; in particular, we have focused on the behavioural, electrophysiological and histopathological findings obtained with different protocols. We addressed the various experimental approaches published to date, by comparing mortality rates, onset of SRSs, neuronal damage, and network reorganization. Based on the evidence reviewed here, we propose that the pilocarpine model can be a valuable tool to investigate the mechanisms involved in TLE, and even more so when standardized to reduce mortality at the time of pilocarpine injection, differences in latent period duration, variability in the lesion extent, and SRS frequency.

## 1 Introduction

The ability to reproduce human diseases in animal models presents a great advantage for modern experimental medicine (Russell, 1964). The ideal animal model is homologous, duplicating the human disorder in every respect. Alternatively, the animal model could be isomorphic, when it duplicates the disorder but not the underlying aetiology (that in many neurological diseases is unknown), or predictive, in the case in which it does not resemble the human disorder but allows predictions about it or its response to treatment (this is the case of the kindling paradigm for epilepsy). A great deal of the knowledge that has improved our understanding of epileptic disorders has derived from appropriate animal models (Purpura et al., 1972; Schwartzkroin, 1983; Pitkänen et al., 2006). This is certainly the case in temporal lobe epilepsy (TLE), the most common type of partial complex seizure in adulthood (Hauser et al., 1996; Wieser, 2004).

The main features of TLE are: (i) the localization of seizure foci in the limbic system, particularly in the hippocampus, entorhinal cortex and amygdala (Bartolomei et al., 2005); (ii) the frequent finding of an “initial precipitating injury” that precedes the appearance of TLE (Mathern et al., 2002); (iii) a seizure-free time interval following the precipitating injury known as “latent period”; and (iv) a high incidence of mesial or Cornu Ammonis (CA) sclerosis, i.e., a unilateral hippocampal lesion leading to atrophy, typically caused by neuronal loss and gliosis in Sommer's sector (the subiculum-CA1 transition zone) and the endfolium (dentate hilus) (Mathern et al., 1997b).

Most of these characteristics can be reproduced in chronic animal models of TLE, particularly kindling or *status epilepticus* (SE) animal models, which come close to being the ideal homologous model mentioned above (Morimoto et al., 2004). The subject of this review, the pilocarpine model, belongs to SE models. This model appears to be highly isomorphic with the human disease, so it has been used in many laboratories since its first description a quarter of a century ago (Turski et al., 1983a,b). Histopathological findings (Covolan and Mello, 2000) in the pilocarpine model and its use in studying the efficacy of antiepileptic drugs (AEDs; Leite et al., 2002) have been reviewed recently. In this paper we will therefore focus on methodological procedures and the behavioural consequences, especially in view of recent advances in this field made possible by an increasing use of video-electroencephalography (EEG) recordings.

Some important features of the pilocarpine model are: (i) the induction of acute SE more rapidly than with intraperitoneal (i.p.) kainic acid, the other convulsant drug commonly used to reproduce TLE in animals; (ii) the presence of a latent period followed by the appearance of spontaneous recurrent seizures (SRSs, chronic phase) (Leite et al., 1990; Cavalheiro et al., 1991); (iii) the occurrence of widespread lesions (see Section 2.3.2), some of them localized in the same brain areas affected in TLE patients, and associated with neuronal network reorganization in hippocampal and parahippocampal regions (for instance, mossy fibre sprouting, interneuron loss and ectopic dentate granule cell proliferation are phenomena shared by TLE patients and pilocarpine-treated animals; Wieser, 2004); (iv) the fact that seizures are poorly controlled by AEDs in patients and pilocarpine-treated epileptic rodents (Glien et al., 2002; Chakir et al., 2006).

The ability of pilocarpine to induce SE is likely to depend on activation of the M1 muscarinic receptor subtype, since M1 receptor knockout mice do not develop seizures in response to pilocarpine (Hamilton et al., 1997). Other cholinomimetics, such as carbachol and oxotremorine, are also able to induce seizures and seizure-induced brain damage when injected either systemically or directly into the brain (Olney et al., 1983; Turski et al., 1983b). In addition, pilocarpine-induced SE can be blocked by systemic administration of the muscarinic antagonist atropine (Clifford et al., 1987). Once seizures are initiated, however, their maintenance depends on other mechanisms since atropine becomes ineffective (Clifford et al., 1987). Experiments in cultured hippocampal neurons have demonstrated that pilocarpine, acting through muscarinic receptors, causes an imbalance between excitatory and inhibitory transmission resulting in the generation of SE (Priel and Albuquerque, 2002). In addition, *in vivo* microdialysis studies have revealed that pilocarpine induces an elevation in glutamate levels in the hippocampus following the appearance of seizures (Smolders et al., 1997). Substantial evidence now supports the suggestion that, following initiation by M1 receptors, seizures are maintained by NMDA receptor activation (Nagao et al., 1996; Smolders et al., 1997).

The pilocarpine model is now widely used and it has been modified in a number of laboratories. Depending on the aims of the experiments, different groups of researchers use different dosages of pilocarpine, pretreatment procedures, animal strain and species. In addition, the duration of SE induced by pilocarpine or the drugs employed to terminate it are often inconsistent. These multifaceted protocols could well contribute to the variable findings obtained in different studies. Indeed, the lack of a standardized approach to the pilocarpine model could play a critical role in how it recreates the human scenario. These aspects have recently led some investigators to criticize the pilocarpine model and to propose alternative approaches to reproduce TLE in animals (Sloviter, 2005; Sloviter et al., 2007). In this review we will compare the different protocols used in the pilocarpine model, and in particular to gain consensus as to what factors may be critical in what is perhaps one of the most appropriate models of TLE.

## 2 Behavioural, electrophysiological and histological features

Injection of pilocarpine induces a SE that is characterized by tonic–clonic generalized seizures. After several hours of SE, pilocarpine-treated animals remit spontaneously and go into a seizure-free period, known as latent period, before displaying the SRSs that characterize the chronic epileptic condition.

### 2.1 *Status epilepticus*

*Behaviour*. The pilocarpine model was developed by Turski's group (1983a,b). These initial studies provided a qualitative but extensive description of the behavioural, electroencephalographic and histopathological consequences of SE induction by pilocarpine. In these experiments, pilocarpine (400 mg/kg, i.p.) was injected in adult male Wistar rats, and a progressive evolution of seizures, similar to that classified by Racine (1972) in the kindling model, was observed.

According to Turski et al. (1983a), animals were motionless for 5–10 min after pilocarpine administration and subsequently displayed oro-facial movements, salivation, eye-blinking, twitching of vibrissae, and yawning. This activity persisted up to 45 min. Then, discontinuous seizures were observed 30 min after injection and lasted up to 90–150 min, before giving way to limbic motor seizures with intense salivation, rearing, upper extremity clonus, and falling. Such seizures were observed every 5–15 min, presenting maximal frequency after 1–2 h. After pilocarpine injection, around 60% of the rats successfully developed SE (Cavalheiro et al., 1991). SE spontaneously remitted 5–6 h after pilocarpine administration, and the animals entered post-ictal coma, lasting 1–2 days. Body weight decreased after SE (10–20%), but recovered to pretreatment values after approximately 1 week (Turski et al., 1989).

**Fig. 1:**
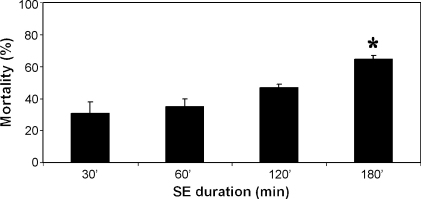
Histogram showing the changes in mortality as function of *status epilepticus* (SE) duration. Values represent the means of three to four different experiments (*n* = 15–20 rats for the different time intervals) in which seizures were quelled by injecting diazepam (20 mg/kg, i.p.) 30, 60, 120 or 180 min after pilocarpine-induced SE. Pilocarpine was used at 380 mg/kg after a scopolamine methylnitrate injection (1 mg/kg, 30 min before pilocarpine) in Sprague–Dawley rats at 8 weeks of age (cf. Biagini et al., 2006, 2008). Note that a 180-min SE causes a significantly (*p* < 0.05) higher mortality when compared with all the other time intervals (analysis of variance followed by Games–Howell test for multiple comparisons).

Mortality rates at the time of the injection have been reported to be around 30–40% for male Wistar rats treated with 300–400 mg/kg pilocarpine; this is a range of doses that is able to reproduce the complete pilocarpine-induced syndrome (Turski et al., 1983a, 1989; Cavalheiro et al., 1991; Liu et al., 1994b). However, other laboratories have found even worse outcomes (Table 1). In an attempt to ameliorate such a high mortality rate, some researchers have divided the pilocarpine treatments, injecting an initial dose of 200 mg/kg, and then additional 100 mg/kg doses until induction of SE (Glien et al., 2001). This repeated administration of low-doses significantly decreased mortality, but even this approach was unsatisfactory as the average delay from the first injection of pilocarpine to the onset of SE could amount up to 360 min, somehow impractical for experimental purposes (Glien et al., 2001). As an alternative approach to reducing mortality, many groups have limited SE duration using diazepam. Indeed, reduction of the SE length does result in a significant decrease in mortality rate (Fig. 1). However, it must be noted that SE duration is crucial for the development of the full syndrome (see below).

**Fig. 2:**
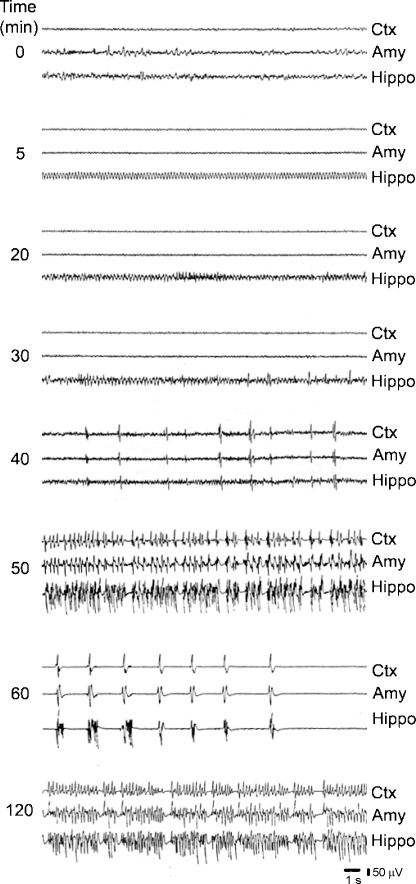
EEG recordings obtained after pilocarpine (400 mg/kg, i.p.) administration. Note that 5 min after injection, low voltage fast activity appears in amygdala (Amy) and neocortex (Ctx), while theta rhythm is evident in the hippocampus (Hippo). Twenty minutes after, high-voltage fast activity is seen in amygdala and neocortex, while spikes superpose in the hippocampus. In the 30 min traces, high voltage spikes are detected first in the hippocampus while at 40 min, high voltage spikes are recorded from all the fields. After 50 min from the injection, electrographic seizures are seen and followed by post-ictal depression (60 min sample). At 120 min, the EEG corresponds to *status epilepticus* (modified from Turski et al., 1983a).

*EEG activity*. EEG recordings during SE immediately after injection have shown that pilocarpine can evoke both ictal and interictal epileptic events as well as that these EEG patterns are correlated with behavioural changes. As shown in Fig. 2, low voltage, fast activity first appears in neocortex and amygdala, while a clear pattern of theta rhythm is evident in the hippocampus. When the behavioural manifestations become more severe, high voltage, fast EEG activity replaces the hippocampal theta rhythm. Moreover, at later stages, animals develop electrographic seizures, characterized by high voltage, fast activity and prominent high voltage spiking that precedes seizures, most likely due to muscarinic system activation (Fisahn et al., 1998; Van der Linden et al., 1999). This activity appears to originate in the hippocampus and to propagate to the amygdala and neocortex (Turski et al., 1983a).

As illustrated in the EEG sample obtained 60 min after pilocarpine injection, the ictal discharges are followed by depression of the EEG activity. By 1–2 h after pilocarpine treatment, the electrographic activity progresses to a SE that lasts 5–6 h, and is followed by gradual normalization of the EEG over several hours. When the animals recover from SE and post-ictal coma (approximately 24–48 h after), the EEG is considered normal although a theta rhythm that is weaker than before pilocarpine treatment can be recorded in the hippocampus (Turski et al., 1983a).

### 2.2 Latent period

The duration of the latent (also termed silent or quiescent) period differs depending on the protocol used. In Wistar rats injected with pilocarpine (380 mg/kg), it lasts from 1 up to 6 weeks with a mean time interval of 14.8 days (Cavalheiro et al., 1991). Varying the pilocarpine dose from 350 up to 400 mg/kg induces only slight differences in the time of onset of SRSs (Liu et al., 1994b). In contrast, when the duration of SE is varied by arresting seizures with diazepam alone or combined with other drugs, such as pentobarbital or phenytoin (Lemos and Cavalheiro, 1995; Fujikawa, 1996; Biagini et al., 2006), the latent period duration changes significantly as a function of the different time intervals of continuous seizure activity in the acute period.

**Fig. 3:**
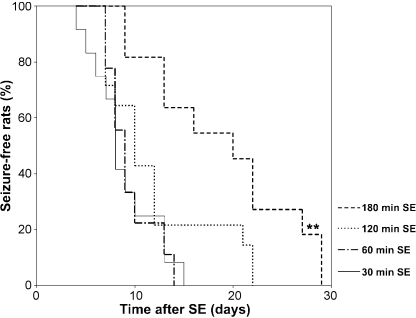
Kaplan–Meier analysis of the time of spontaneous seizure appearance after *status epilepticus* (SE) induced by injecting Sprague–Dawley rats with pilocarpine (380 mg/kg, i.p.) after a scopolamine methylnitrate injection (1 mg/kg, 30 min before pilocarpine). Note that the spontaneous seizure onset is progressively delayed by increasing SE duration (seizures were quelled by injecting 20 mg/kg diazepam at different time intervals from the pilocarpine injection, cf. Biagini et al., 2006). The log rank test revealed a significant (**p < 0.01) difference between the 180-min SE group and the others (*n* = 9–14/group).

According to Lemos and Cavalheiro (1995), rats undergoing 30-min long SE and treated with a single (i.p.) injection of diazepam (10 mg/kg) and pentobarbital (30 mg/kg) do not develop SRSs. In contrast, animals presenting with SE lasting 1, 2, 6 or several hours exhibit latent periods of 52, 38, 17 and 14 days, respectively. However, we have been unable to confirm these data since we found that the latent period is progressively shortened by decreasing the SE length (Fig. 3) (Biagini et al., 2006). It should also be noted that a recent study based on continuous video-EEG recordings, has shown a mean latent period of approximately 7 days after a 2 h SE (Goffin et al., 2007). In these studies, Lemos and Cavalheiro (1995) used Wistar rats weighing 200–250 g, whereas Goffin et al. (2007) analyzed Wistar rats with a body weight range of 285–350 g that was similar to that of the Sprague–Dawley rats (270–300 g) used by Biagini et al. (2006). Thus, the differences found by these authors are not explained by the rat strain or age. In contrast, a possible factor of variance is the pharmacological approach to abort SE, since the animals developing SRSs more rapidly were treated only with diazepam (20 mg/kg, i.p.) (Biagini et al., 2006; Goffin et al., 2007).

Apart from occasional rats that continue to present seizures in the first 3 days after SE (Cavalheiro et al., 1991), pilocarpine-treated animals generally show normal behaviour and EEG activity during the latent period. It is, however, believed that during the latent period several pathophysiological phenomena that are related to epileptogenesis may occur. These events include mossy fibre sprouting, interneuron loss, rewiring of synaptic circuits, glial cell activation, and ectopic cell proliferation (Dalby and Mody, 2001; Pitkänen and Sutula, 2002). As there is no definitive evidence that any of these phenomena is critical for epileptogenesis, it will be important to evaluate in future studies how they vary in relationship to SE duration and the consequent alterations in the latent period extension. For instance, we have recently shown a direct relation between the latent period duration and the extent of induction in glial cells of the rate-limiting enzyme of the neurosteroid pathway, the cholesterol side-chain cleavage enzyme coupled to the cytochrome P450 (P450scc) (Biagini et al., 2006). Neurosteroids such as allopregnanolone are anticonvulsants that are mainly synthesized in astrocytes; these glial cells are highly activated by seizures and brain damage. Interestingly, we have found that a longer SE corresponds to a larger hippocampal lesion and thus a stronger activation of glial cells that finally might be able to extend the latent period by synthesizing seizure-modulating substances like neurosteroids (Biagini et al., 2006, 2008).

### 2.3 Chronic period

The chronic period follows epileptogenesis and is characterized by the appearance of SRSs. As with the latent or silent phase, discrepancies in the characteristics of the chronic period emerge from studies made in different laboratories with the pilocarpine model. We will therefore discuss these discrepancies by considering first the data regarding the seizure characteristics, and then those concerning the neuronal damage that occurs in the brain of these animals.

#### 2.3.1 Chronic limbic seizures

*Behaviour*. SRSs, which appear in pilocarpine-treated rats after the latent period, have recently been reclassified by Veliskova (2006) according to the following criteria: 1, staring and mouth clonus; 2, automatisms; 3, monolateral forelimb clonus; 4, bilateral forelimb clonus; 5, bilateral forelimb clonus with rearing and falling; 6, tonic-clonic seizure. As proposed by Goffin et al. (2007), this classification could be simplified by referring to criteria 1–3 as partial seizures, and 4–6 as secondarily generalized seizures. According to this classification, SRSs begin approximately 7 days after SE as partial seizures, becoming generalized seizures in the following days (Goffin et al., 2007). Arida et al. (1999) performed a careful analysis of SRSs in adult male Wistar rats injected with 350 mg/kg pilocarpine, and found a relationship between the rate of occurrence of seizures and the duration of the latent period. They detected the first spontaneous seizure between 3 and 30 days after pilocarpine injection. Shorter latent periods (3–5 days) were found in animals that eventually experienced more (124–727) SRSs; longer latent periods (28–30 days) were related to lower number of SRSs (range 45–584) during 135 days of observation.

An additional feature of SRSs is that, once manifested, they appear to recur in a clustered way in a cycle peaking every 5–8 days (Goffin et al., 2007) or more (Arida et al., 1999). Besides these observations, SRSs appear to be relatively regular throughout the lifetime of the animal (Priel et al., 1996). It should also be emphasized that seizure frequency has been found to vary according to the method used to monitor the animals (visual inspection, video monitoring or continuous video-EEG recording) and the type of seizures scored (partial or generalized). Originally, in adult male Wistar rats given 300–320 mg/kg of pilocarpine, a mean latent period of 14 days and a frequency of 2.8 seizures per week were found by continuously video monitoring (Cavalheiro et al., 1991, 1996; Lemos and Cavalheiro, 1995; Arida et al., 1999). Seizure frequency was higher during the diurnal period (from 7:00 a.m. to 7:00 p.m.) when the animals were kept in a 12-h dark/light cycle (Arida et al., 1999). Similarly, video-EEG recordings have established that approximately 67% of daily seizures occur during the light phase of the cycle (Goffin et al., 2007). Arida et al. (1999) have also reported that animals presenting with a low SRS occurrence (mean = 8) during the first 15 days, reach a peak (75 SRSs) at day 76. Then, they decrease to 61 at day 105 and do not display any significant change between days 120 and 135. Animals that are characterized by higher rates of SRSs (*n* = 29) during the first 15 days, reach a peak (51 SRSs) at day 60 and do not change significantly between days 75 and 135.

*EEG activity*. SRSs in pilocarpine-treated animals are characterized by bursts of spiking activity in the hippocampus that spread to the neocortex in 90% of the cases (Cavalheiro et al., 1991). Electrographic seizures rarely last more than 60 s and are followed by depressed background activity with frequent EEG interictal spikes. Bursts of spiking activity are not observed in the neocortex alone (Cavalheiro et al., 1991). In addition, interictal activity is more intense when animals are seizure-free and during the sleep period while they are almost undetectable during motor activity and paradoxical sleep (Arida et al., 1999).

#### 2.3.2 Seizure-induced damage and network reorganization

**Fig. 4:**
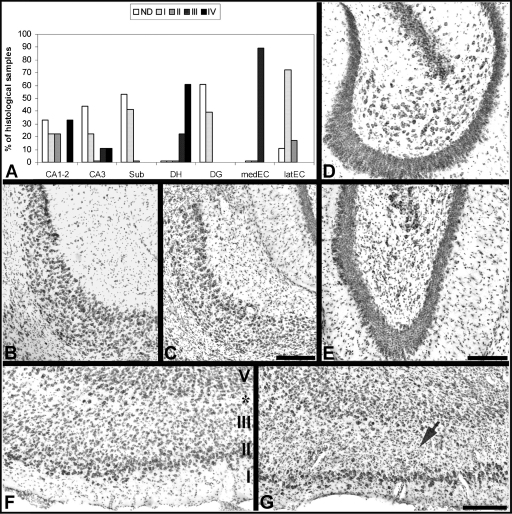
Grading of neuronal damage in the hippocampal formation of pilocarpine-treated rats performed in toluidine blue-stained sections that were cut 3 weeks after the pilocarpine injection. (A) grading of the lesion (*n* = 18) from non-detectable (ND) to almost complete lesion (grade IV) is shown for different areas (see Biagini et al., 2005 for the complete description of the method). Sections of an intact subiculum (B) and of a grade II-damaged subiculum (C). Non-epileptic control (NEC) (D) and pilocarpine-treated (E) dentate gyrus; note that the section in (E) presents a grade IV lesion in the hilus, but a well-preserved granule cell layer. Medial entorhinal cortex close to the boundary with the lateral entorhinal cortex in control (F) and pilocarpine-treated rats (G). Note in (G) the reduced thickness of layer III, in which neurons are replaced by a glial infiltrate (arrow). In (F) and (G), apart the sparse layer IV located immediately above the lamina dissecans (asterisk), the other cortical layers are indicated at the boundary with the parasubiculum. Scale bars for panels (B) and (C), (F) and (G) are 250 μm; for (D) and (E) is 200 μm. Abbreviations in this figure: CA1–2, CA3: Cornu Ammonis hippocampal subfields; DG: dentate gyrus; DH: dentate hilus; medEC: medial entorhinal cortex; latEC: lateral entorhinal cortex; Sub: subiculum.

Animals experiencing SE for several hours (i.e. not terminated by any pharmacological intervention) show histopathological alterations that are localized within the olfactory cortex, amygdala, thalamus, hippocampal formation, and neocortex. Several areas appear swollen and edematous and many cells are dark and shrunken (Turski et al., 1983a). Some areas such as the lateral thalamic nucleus, substantia nigra and dentate hilus are damaged only in animals that develop chronic SRSs (Priel et al., 1996). However, other authors have found that hilar mossy cells can survive SE and be subsequently activated by SRSs (Scharfman et al., 2001). Cell loss (Fig. 4) has been detected in the subiculum (Knopp et al., 2005; de Guzman et al., 2006), amygdala (Turski et al., 1983a) and layer III of the medial entorhinal cortex (Du et al., 1995; Biagini et al., 2005; Wozny et al., 2005). In addition, injured neurons, mainly interneurons, can be found in the hippocampus (CA1 and CA3 stratum pyramidale and radiatum), amygdala and piriform cortex (Mello and Covolan, 1996).

It has been reported that superficial layers of the sensorimotor cortex are particularly affected with marked atrophy and dendritic sprouting, suggesting lesion, reorganization and neuroplasticity of neocortical networks (Sanabria et al., 2002). The thickness of neocortex is indeed reduced in pilocarpine-treated animals compared to controls. In addition, the laminar cortical organization is disrupted, and a lower neuronal density is evident in epileptic animals. Reactive gliosis is also observed in the superficial third of the cortex of epileptic animals (Silva et al., 2002).

Network reorganization in pilocarpine-treated animals occurs presumably as consequence of neuronal loss and SE-induced sprouting (Lehmann et al., 2001). SE also appears to induce formation of ectopic cells that are recruited into the nascent network (Scharfman et al., 2003). Neocortical neurons with a dense arborisation of apical dendrites in the most superficial layers are apparent in rats with SRSs (Silva et al., 2002). At variance, the presence of subicular neurons with a reduced arborisation and spine density in the proximal part of the apical dendrites suggests a partial deafferentiation from CA1 (Knopp et al., 2005). It is still debated whether and how such changes participate in the initiation of SRSs, and how they lead to chronic epilepsy. Indeed, studies by Mello and collaborators (Longo and Mello, 1997; Dos Santos et al., 2000; Longo et al., 2002) have shown that cycloheximide administered to pilocarpine-treated rats to block protein synthesis, and thus mossy fibre sprouting, causes little change in latent period duration and no consequences for the establishment of a chronic epileptic condition. However, these findings have been questioned by another study in which cycloheximide treatment failed to reduce mossy fibre sprouting (Williams et al., 2002).

The contribution of neuronal damage to the development of SRSs is also unclear. It is well known that mossy fibre sprouting can be observed without neuronal damage (i.e., in the kindling model; cf. Morimoto et al., 2004) and that electroconvulsive shock seizure repeated for several days induces mossy fibre sprouting without cell loss (Gombos et al., 1999). Intriguingly, it has also been proposed that epileptogenesis may occur without neuronal cell loss (Zhang et al., 2002). In these experiments, kainic acid-pilocarpine-treated epileptic rats did not exhibit mossy fibre sprouting.

## 3 Pilocarpine doses

Behavioural alterations induced by pilocarpine are known to be dose-dependent. A number of studies have compared doses of pilocarpine ranging from 100 to 400 mg/kg in adult male Wistar rats (Turski et al., 1983a; Liu et al., 1994b). Irrespective of the dose, the animals first became immobile, and this was followed by gustatory and olfactory automatisms (salivation, oro-facial movements, and vibrissae twitching). Subsequently, animals experienced limbic motor seizures, but only with the highest dose of pilocarpine (400 mg/kg) (Turski et al., 1983a; Cavalheiro et al., 1987; Liu et al., 1994b). Higher doses resulted in a greater likelihood of induction of the complete syndrome and reduced latency to SE, but also increased mortality rate (Clifford et al., 1987; Liu et al., 1994b). In Sprague–Dawley rats pilocarpine 400 mg/kg induced SE in 83% of animals and mortality was 100% (Jope et al., 1986).

Alterations in EEG recordings have also been reported to be dose-dependent, occurring in two stages. Low voltage fast activity first appeared in the cortex and amygdala, concurrent with theta rhythm in the hippocampus. High voltage fast activity then superseded hippocampal theta, and isolated high voltage spikes were initially registered exclusively in the hippocampus. In animals treated with low doses of pilocarpine (100–200 mg/kg) this scenario persisted up to 2 h, and the normal EEG profile recovered immediately after. Animals receiving the highest dose of pilocarpine (400 mg/kg) exhibited electrographic seizures that were preceded by high voltage fast activity and prominent high voltage spiking, followed by variable periods of depression (Fig. 2). These changes originated in the hippocampus and rapidly spread to the amygdala and neocortex. Electrographic activity progressively built up into a SE after 1–2 h. This pattern persisted for 5–6 h and was followed by a progressive normalization. Twenty-four hours later, animals receiving the highest dose of pilocarpine exhibited residual theta rhythm in the hippocampus (Turski et al., 1983a).

Neuropathological consequences were also different in animals that were treated with different doses of pilocarpine. Damage, which was confined to piriform cortex and anterior olfactory nuclei when rats were injected with the lowest dose of pilocarpine (100 mg/kg), extended to amygdala, cortical and basal nuclei, with 200 mg/kg, although limbic motor seizures were not observed. Rats that display severe limbic motor seizures after 200 mg/kg, showed additional damage in medio-dorsal thalamic nuclei and in neocortex. Injection of pilocarpine 400 mg/kg resulted in the profound neuropathological alterations already described in the previous section (see also Turski et al., 1983b).

## 4 Routes of administration of pilocarpine

It has been demonstrated that intra-hippocampal pilocarpine administration is as efficient as the i.p. route in inducing SE and then chronic epilepsy. In fact, animals receiving pilocarpine directly into the hippocampus show not only the same behavioural, electrographic and neuropathological alterations exhibited by those treated systemically, but have the advantage of a drastically reduced mortality (Furtado et al., 2002). Indeed, the ratio of animals developing SE and surviving after the intra-hippocampal injection has been found to be higher (71%) than with systemic administration. In Wistar rats injected intra-hippocampally with pilocarpine (2.4 mg/μl) in 1 μl, the first epileptiform discharge occurred in the hippocampus and spread to the amygdala, and was associated with oro-facial automatisms. Epileptiform discharges observed during SE were characterized by clonic movements of the head and forelimb that were mirrored by a larger amplitude signal in amygdala than in hippocampus. Approximately 70% of the animals experienced SE within 30 min after the intra-hippocampal injection and presented SRSs after a latent period of 2–30 days. All rats showed positive neo-Timm staining in the internal molecular layer of the dentate gyrus, indicating mossy fibre sprouting. In addition, in some of these animals there was stronger staining in the ventral hippocampus ipsilateral to the site of pilocarpine injection (Furtado et al., 2002).

Pilocarpine is used also *in vitro* to induce epileptic activity in hippocampal neuronal networks. In rat brain entorhinal cortex-hippocampal slices, pilocarpine at 10 μM was able to induce an electrographic activity characterized by different types of epileptiform activity: (i) one type, resembling the interictal activity observed in epileptic patients when not presenting with seizures, occurred at 0.2–0.4 Hz simultaneously in the entorhinal cortex and hippocampus; (ii) a second type of interictal discharge only occurred in the hippocampus at 0.6–3.8 Hz; (iii) the third type was an ictal-like discharge recorded in both entorhinal cortex and the hippocampus, lasting 4–18 s. Analysis of the time delay demonstrated the onset of ictal discharges in the entorhinal cortex, from where they propagated to the dentate area and, finally, to the hippocampus (Nagao et al., 1996). These results were obtained at a low extracellular potassium concentration (3.25 mM). In contrast, Marchi et al. (2007) were unable to induce any seizure-like activity when applying acutely pilocarpine on rat hippocampal slices in presence of 4.35 mM potassium, whereas seizures occurred by increasing potassium up to 6 mM. However, Marchi et al. (2007) used slices obtained in the coronal plane at dorsal hippocampal levels, instead of the horizontal entorhinal cortex-hippocampal slices studied in the previously described experiment (Nagao et al., 1996). Pilocarpine has also been shown to induce bursting activity in CA1 pyramidal neurons in organotypic hippocampal cultures, a model in which other molecular features of pilocarpine-induced changes were equally reproduced (Poulsen et al., 2002).

## 5 Duration of *status epilepticus*

It has been shown that the duration of SE during the initial insult is critical to the development of SRSs and brain damage. However, the different protocols of drug treatment used in different studies have resulted in contrasting findings. The first study to address the effects of SE duration was conducted in adult male Wistar rats injected with a single dose of pilocarpine (300–320 mg/kg, i.p.) followed by a combined diazepam (10 mg/kg) and pentobarbital (30 mg/kg) treatment (Lemos and Cavalheiro, 1995). This study reported a progressive increase in the mean latency to the first spontaneous seizure and a decrease in seizure frequency in animals with shorter SE (1 and 2 h). In addition, these animals presented less severe neuropathological alterations (Lemos and Cavalheiro, 1995). Finally, this study also showed that there was a minimum duration of SE prerequisite for the development of chronic epilepsy; animals experiencing only 30 min of SE did not develop SRSs.

These findings are at odds with our data (Fig. 3) as well as with those of Klitgaard et al. (2002), who have observed SRSs in rats that had experienced 30 min SE. The latent period in these animals lasted 3 weeks and they presented with brain lesions. In animals that experienced 1 h of SE, disruption of the hippocampal pyramidal layer was also observed (Klitgaard et al., 2002). In some cases, cells were severely shrunken and darkened. Extensive pathological alterations were found in the hippocampus, basal amygdaloid nucleus, dorso-medial thalamic nucleus, substantia nigra, parietal and temporal neocortex, piriform and entorhinal cortex of animals that underwent longer SE (up to 6 h), indicating that the severity of neuropathological changes increased with increased SE duration. Similar observations have been reported by others (Liu et al., 1994b; Lemos and Cavalheiro, 1995). In Klitgaard et al. (2002) experiments, SE was terminated by injecting diazepam intravenously at 10 mg/kg, while other authors generally used the i.p. route and doses ranging from 5 mg/kg (Wozny et al., 2005) up to 20 mg/kg (Goffin et al., 2007), with single or repeated injections. In particular, Lemos and Cavalheiro (1995) used a combination of diazepam and pentobarbital, which could be more efficient in terminating SE (Morrisett et al., 1987).

## 6 The lithium–pilocarpine model

Variations of the pilocarpine model have been established by combining this convulsant with other drugs, such as lithium (3 mEq/kg in 0.1–0.2 ml of saline, s.c.; Honchar et al., 1983), picrotoxin (0.5–2.0 mg/kg, i.p.; Hamani and Mello, 1997, 2002), cycloheximide (1 mg/kg, s.c.; Longo and Mello, 1997; Longo et al., 2002), MK-801 (Hughes et al., 1993) and N omega-nitro-l-arginine methyl ester (1–125 mg/kg, i.p.; Starr and Starr, 1993). The lithium–pilocarpine combination has been the most widely used. Lithium is extensively used as mood stabilizer in maniac-depressive illness, and it has been more recently introduced in the treatment of acute brain injuries and chronic neurodegenerative disease (Wada et al., 2005).

Lithium is generally administered 24 h before the SE induction, and it allows a conspicuous reduction of the pilocarpine dose required to induce seizures (30 mg/kg). The sequence of behavioural changes observed in animals undergoing a SE was very similar for lithium–pilocarpine compared to pilocarpine administered alone: it was characterized by staring, head bobbing, blinking and wet-dog shakes; seizures subsequently appeared 30 min later, each lasting approximately 30–45 s and recurred every 2–5 min (Honchar et al., 1983; Jope et al., 1986; Clifford et al., 1987). It has been reported that animals treated with lithium + pilocarpine showed a decreased variability in time to SE onset (Clifford et al., 1987).

Staring observed in lithium–pilocarpine-treated animals was associated with single spikes in the EEG, followed by generalized spike activity separated by intermittent low voltage activity, after which spike trains became continuous (Jope et al., 1986). In some animals, the ventral forebrain (ventral globus pallidum and nucleus accumbens) was the apparent site of origin for electrographic seizures that then spread to other areas. In other animals, electrographic seizures began simultaneously at multiple sites (Clifford et al., 1987). However, there were no differences concerning the presence of a single *versus* multiple sites of origin in lithium–pilocarpine and high-dose pilocarpine treatment groups (Clifford et al., 1987).

Neuronal damage resulting from SE was essentially similar in the lithium–pilocarpine and high-dose pilocarpine models (Clifford et al., 1987). Thus, the syndromes produced by lithium–pilocarpine and high-dose pilocarpine treatments are behaviourally, metabolically, electrographically and neuropathologically indistinguishable. The major apparent difference seems to be in the increased sensitivity to pilocarpine in lithium-pretreated rats (Clifford et al., 1987). Also, the rate of success in developing tonic–clonic seizures and SE after lithium pretreatment is 100%, an improvement when compared with what is seen by employing high-dose of pilocarpine where it is around 60% (Goffin et al., 2007). André et al. (2007) have recently characterized the time course of the neuropathological changes occurring in magnetic resonance imaging (MRI) of the brain of lithium–pilocarpine-treated rats. In this study, early changes in MRI scans were first detected in the piriform cortex, entorhinal cortex, thalamus and amygdala 6 h after SE. The hippocampus appeared to be affected only 36–48 h later; these hippocampal alterations appeared, however, to intensify progressively until 80 days after SE. MRI changes also reappeared in the parahippocampal cortex with SRS onset.

Unfortunately, in the lithium–pilocarpine model the mortality rate remained very high (92% in Jope et al., 1986; >95% in Morrisett et al., 1987) when pilocarpine was given in a dose of 30 mg/kg (see Table 1). Decreasing the dose of pilocarpine in lithium-treated rats resulted in a decrease in mortality rate, but this was concomitant with a fall in the success rate for inducing SE (both at 50%; Jope et al., 1986). Neither lithium (3 mEq/kg) nor pilocarpine (30 mg/kg) caused abnormal EEG responses when administrated alone (Jope et al., 1986; Clifford et al., 1987). Additionally, inverting the order of pilocarpine and lithium administration resulted in no seizures, although co-administration of the agents produced seizures in three out of five rats. Finally, lithium pretreatment is effective only when pilocarpine is administered within 24 h; in fact, the animals did not display seizures when pilocarpine was given 48 h after lithium (Clifford et al., 1987).

A further modification of the lithium pilocarpine protocol has also been proposed. Glien et al. (2001) administered lithium followed by pilocarpine 24 h later. If pilocarpine was administered as a single dose of 30 mg/kg and SE duration was limited to 90 min, mortality was 45%, and 80% of survivors developed SRS. However, if pilocarpine was given in divided doses of 10 mg/kg at 30 min intervals until SE ensued, mortality was reduced to 7%, and 85% of animals developed SRS. One of us (RSGJ) has been using a further modification of this protocol in which the divided doses of pilocarpine have been reduced to 5 mg/kg. In addition, when animals develop SE, they are administered a low dose of the central muscle relaxant, xylazine, which reduces the severity of clonic muscle contractions without affecting electrographic seizures (see Yang et al., 2006; Thompson et al., 2007). Using this approach we have been able to routinely obtain mortality rates of zero in groups of both rats and C57BL/6 mice (Woodhall, G.L. and Jones, R.S.G. unpublished results) with a 90–100% success in generating SRSs.

## 7 Pilocarpine in combination with other drugs

### 7.1 Pretreatments

#### 7.1.1 Anticholinergic drugs

Administration of the muscarinic antagonist, atropine (1–5 mg/kg, s.c.), to lithium-pretreated rats 30 min prior to pilocarpine prevented the induction of SE along with cell damage (Clifford et al., 1987; Morrisett et al., 1987). When pretreatment time is reduced to 15 min, seizure activity was induced in 75% of the animals (Jope et al., 1986). Administration of atropine methylbromide (5 mg/kg, i.p., 20 min prior to pilocarpine) has been used to block the peripheral cholinergic side effects of pilocarpine, without interfering with the development of SE and chronic seizures (Kobayashi et al., 2003; Kumar and Buckmaster, 2006; Kwak et al., 2006). The antimuscarinic drug, alpha-methylscopolamine does not cross the blood brain barrier so it cannot interfere with the central actions of pilocarpine (Clifford et al., 1987). Alpha-methylscopolamine administered 30 min prior the pilocarpine has therefore been widely used to minimize peripheral cholinergic activation. Without this pretreatment, animals exhibit classic signs of peripheral cholinergic stimulation, including piloerection, salivation, tremor, chromodacryorrhea and diarrhea after pilocarpine injection (Clifford et al., 1987). There is no evidence that low doses (1 mg/kg) of alpha-methylscopolamine alter central effects of pilocarpine, but higher doses (i.e., 10 mg/kg in mice and 20 mg/kg in rats, s.c.) can prevent induction of SE (Turski et al., 1983a,b, 1984).

#### 7.1.2 Antiepileptic drugs

Diazepam (5 or 10 mg/kg) prevents the development of behavioural and EEG alterations induced by pilocarpine as well as the subsequent neuropathological alterations in both lithium-pretreated and high-dose pilocarpine rats (Turski et al., 1983a; Jope et al., 1986; Morrisett et al., 1987) or mice (Turski et al., 1984). An extended study of the effects induced by different classes of drugs able to affect seizure onset has been reported by Morrisett et al. (1987). This study showed that phenobarbital (32.5 mg/kg), carbamazepine (100 mg/kg) and paraldehyde (0.3 mg/kg) were able to prevent SE when administered 15 min prior to pilocarpine. Phenytoin (200 mg/kg) prevented SE in two out of a total of three rats and prolonged the latency to seizure in the remaining rat. Sodium valproate (300 mg/kg) increased the latency to seizure initiation but was ineffective in preventing SE (Morrisett et al., 1987).

### 7.2 Post-treatments

#### 7.2.1 Anticholinergic drugs

The ability of a number of drugs to halt pilocarpine-induced SE has been tested. Muscarinic receptor antagonists are generally ineffective in terminating seizures once SE is established. Atropine, injected 20 min following pilocarpine in lithium–pilocarpine-treated rats did not alter SE development and all animals subsequently died (Morrisett et al., 1987). This finding is in agreement with results obtained in patients suffering SE, in which anticholinergics were also ineffective (Jope et al., 1986). In contrast, administration of atropine, around the time when forelimb clonus starts, terminated seizures and all animals survived (Jope et al., 1986; Clifford et al., 1987). Similar results were obtained with scopolamine (10 mg/kg), which failed to prevent or reduce pilocarpine-induced seizures in mice when injected after high dose pilocarpine (Turski et al., 1984).

#### 7.2.2 Antiepileptic drugs

SE is a life-threatening clinical emergency in human patients and efficacious treatments are required to treat it. AEDs for treatment of SE have largely been tested in the pilocarpine model. Morrisett et al. (1987) has shown that diazepam (20 mg/kg), phenobarbital (32.5 mg/kg), phenytoin (100 mg/kg), valproate (300 mg/kg) and carbamazepine (100 mg/kg) are unable to halt SE in lithium–pilocarpine-treated rats. In contrast, paraldehyde (0.3 mg/kg, intramuscular) is effective. The efficacy of paraldehyde treatment has been confirmed recently by others (Kubová et al., 2005).

Jones et al. (2002) have suggested that diazepam is ineffective when administered 15 min after SE is initiated, because animals develop pharmacoresistance. On the other hand, it has been found that diazepam (10 mg/kg), co-administered with pentobarbital (30 mg/kg; Lemos and Cavalheiro, 1995) or with phenytoin (60 mg/kg; Fujikawa, 1996) can terminate both motor and electrographic seizures in pilocarpine-treated rats. However, 2 h after diazepam plus pentobarbital treatment, the EEG returned to normal only in those animals subjected to a SE of 1 h or less. SEs of longer duration were associated with longer recovery periods. Animals, with SE lasting longer than 1 h, later developed SRSs and morphological alterations typical of the chronic model (Lemos and Cavalheiro, 1995). Interestingly, Goffin et al. (2007) have reported that diazepam (20 mg/kg) administered after 2 h of SE was able to terminate electrographic seizures in 3–4 h.

It appears that diazepam is less effective in terminating motor seizures compared to other drugs, such as paraldehyde (Morrisett et al., 1987; Biagini et al., 2001; Kubová et al., 2005), or to combinations of diazepam with barbiturates (Lemos and Cavalheiro, 1995; Fujikawa, 1996; Biagini et al., 2001). Nevertheless, a single diazepam injection (20 mg/kg, i.p.) 30, 60, 120 or 180 min after the SE onset elicited significant differences in mortality and SRSs depending on SE duration (Figs. 1 and 3). When diazepam was not applied, SE remitted spontaneously within few hours after pilocarpine treatment (Lemos and Cavalheiro, 1995).

## 8 Phylogenetic characteristics

### 8.1 Species dependency

Rats and, to a lesser extent, mice represent the animal species used most frequently to reproduce this animal model of TLE. The acute behavioural and EEG features of pilocarpine-induced seizures are generally similar in these two species. Following SE induced by pilocarpine (340 mg/kg), adult (25–30 g) male albino mice, mortality rate was 25–50%, mean latent period was 14.4 days, SRSs lasted 50–60 s and they occurred with a frequency of 1–5 seizures per week (compare Cavalheiro et al., 1991 *vs.* 1996 and Turski et al., 1984). It should be emphasized that mice and rats show similar dose-dependent responses to pilocarpine; in addition, in both cases only the highest doses of pilocarpine were able to induce recurrent SRSs at a later stage. However, mice may be more sensitive to pilocarpine because the dose needed to provoke seizures is very close to the lethal dose (Turski et al., 1983a, 1984). In fact, contrary to the situation in rats, even the lowest dose of pilocarpine (100 mg/kg) was unable to induce behavioural alterations in every mouse tested. Mice receiving 200–300 mg/kg of pilocarpine were motionless, displayed body tremor and occasionally showed myoclonus of hindlimbs. Following 325 mg/kg, myoclonic seizures could be rarely observed. However, clonic–tonic seizures leading to death were observed in 25% of animals. Mortality increased to 50% and 100% with 350 and 400 mg/kg of pilocarpine, respectively. Furthermore, motor limbic seizures appeared more rapidly in the animals treated with the highest pilocarpine dose.

Progressive damage to various brain regions with increasing doses of pilocarpine have been observed in mice as in rats. Low doses of pilocarpine elicited minor behavioural and EEG changes without inducing detectable pathological alterations. Intermediate doses (200 mg/kg) caused mild damage largely confined to the piriform cortex and olfactory nuclei, whilst with the highest doses (300, 325 and 350 mg/kg) consistent and long-lasting behavioural and EEG changes (including motor limbic seizures and limbic SE) and extensive pathological alterations (localized in the olfactory cortex, thalamus, amygdaloid complex, hippocampal formation, neocortex and substantia nigra) were observed (Turski et al., 1984).

Pilocarpine has been tested as a convulsant in other species. In the guinea pig “whole-brain” preparation, seizures have been evoked by millimolar concentrations of pilocarpine or by breakdown of blood brain barrier (Uva et al., 2008). Similar findings were reported by Marchi et al. (2007), suggesting that pilocarpine in this model is not epileptogenic *per se*, but it needs the presence of cofactors able to increase neuronal excitability. In particular, Marchi et al. (2007) reported increased blood brain barrier permeability as well as enhanced production of inflammatory mediators, such as interleukin-1β, in rats treated systematically with pilocarpine. In contrast, pilocarpine was successful in immature rabbits and results of these experiments will be discussed in the following chapter on pilocarpine and ontogenesis (see Section 9).

### 8.2 Strain dependency

Most studies have been carried on Wistar rats. However, results from Sprague–Dawley rats have not shown major differences in the development of behavioural and electrographic alterations (Honchar et al., 1983; Jope et al., 1986), while ameliorating the mortality rates (Table 1). Pilocarpine effects were instead more pronounced in Long-Evans rats, in which a higher mortality rate, larger damage to the hippocampus and a behavioural outcome worse than in Wistar rats was found (Hort et al., 2000).

Shibley and Smith (2002) have conducted a detailed study comparing the pilocarpine model in two different mouse strains. They showed that SE in C57BL/6 inbred mice was delayed compared to CD-1 outbred mice. However, the mean number of seizures, the time to onset of SRSs and the frequency of SRSs were similar. Mice of either strain that experienced less than 3 seizures after the injection did not develop chronic epilepsy. In addition, the frequency of SRSs was related to the seizure frequency exhibited 2 h after the pilocarpine treatment for both CD-1 and C57BL/6 mice. Robust mossy fibre sprouting in the inner molecular layer was observed in all animals that developed spontaneous seizures. Cresyl violet staining of brain tissue from CD-1 and C57BL/6 mice suggested that the distribution of cell loss in the temporal hippocampus was similar to that observed in rats and in albino mice (Shibley and Smith, 2002).

A different profile of responses to pilocarpine injection was found in mice belonging to different inbred strains (Table 2). According to Winawer et al. (2007), mice belonging to the A/J strain more frequently developed SE, although with a longer latency, when compared to DBA mice. Differences were also found in the survival rate of these different strains: DBA survived to SE but presented higher mortality in the chronic period, while A/J mice were less able to cope with SE and the mortality rate was found to be dose-dependent by varying pilocarpine from 200 to 300 mg/kg. Neuronal cell loss was more pronounced in the CA1 and CA3 pyramidal cell layer of DBA mice, which presented also enhanced proliferation of ectopic granule cells. In another work (Chen et al., 2005), the inbred mouse strain FVB/N was compared with C57BL/6 and CD-1, demonstrating a better response in terms of successful development of SE (FVB/N *vs.* CD-1) but a worse outcome for mortality (FVB/N *vs.* C57BL/6). In general, FVB/N mice presented larger hippocampal damage and, curiously, a stronger reactive gliosis.

## 9 Ontogenetic characteristics

Evidence obtained from different studies shows that behavioural and EEG changes following systemic administration of pilocarpine to rats (Cavalheiro et al., 1987; Priel et al., 1996) are age-dependent. There is, however, controversy over the minimum age needed to develop the full syndrome for a chronic model of epilepsy. In addition, there are concerns on the validity of modeling seizures in the first 2 weeks postpartum, as in this period the hypothalamo–pituitary–adrenal axis is immature in rodents (Sapolsky and Meaney, 1986; Vazquez, 1998) but not in humans (Gunnar and Donzella, 2002). Thus, plasma corticosterone levels, which are still elevated at P1, decrease to a minimum at P3 and are not increased by stress stimuli until P12 (Sapolsky and Meaney, 1986). Glucocorticoids are known to enhance the lesion caused by several agents in the hippocampus (Sapolsky, 2000). Metyrapone, an inhibitor of glucocorticoid production, was shown to reduce loss of pyramidal cells in CA1 after kainic acid induction of SE (Smith-Swintosky et al., 1996). In agreement with these findings, P10 rat pups presenting with higher corticosterone levels developed more cognitive defects after SE induced by administration of lithium–pilocarpine (Lai et al., 2006).

A number of studies have investigated seizure effects from the first week postpartum until puberty. Cavalheiro et al. (1987) demonstrated that pilocarpine (100–380 mg/kg)-induced hypoactivity, head and whole body tremors, loss of righting reflex and scratching movements for up to 90 min in 3–6-day-old Wistar rats. There was no evident relationship between dose and behavioural changes. The same doses induced in 7–12-day-old rats more complex changes that lasted for 1–2 h. In animals at P11-12, pilocarpine (200 mg/kg) induced forelimb clonus, head bobbing and loss of postural control and motor seizures in 11 out of 16 rats, 4 of which evolved into SE. At P24–60, rats presented with the same profile as adult animals (cf. Turski et al., 1983a; Liu et al., 1994a). However, the latent period was shorter in older animals (Priel et al., 1996).

Mortality also appears to be age-dependent. In Wistar rats it was high between P15 and 21 (when the success rate in inducing SE is higher) and lower in younger (when no SE is inducible) or older animals (Cavalheiro et al., 1987). Priel et al. (1996) have also reported a higher mortality rate in rats at P18 and 35, when young animals had a better probability of developing SRSs. Rat pups (P7–17) presented lower mortality rates but did not develop SE and SRSs; peripubertal and pubertal rats gradually approximated the mortality rate observed in adults and more frequently became epileptic (Priel et al., 1996). According to another report, increase in seizure susceptibility occurred primarily after P100 (Patel et al., 1988). In contrast, Ferreira et al. (2003) observed permanent absence-like epilepsy in adult rats that experienced pilocarpine-induced SE between P7 and 17 days; they found no difference in the behavioural responses, EEG and the spontaneous seizures observed in groups of different age. Spike-and-wave discharges occurred bilaterally in the neocortex, concomitantly or not with staring, and occasionally with clonic movements or single jerks (Ferreira et al., 2003).

Electrographic changes display more complex patterns in older *versus* younger rats. Pilocarpine (380 mg/kg) induced flattening of background activity at P3–6 and electrographic seizures at P11–12. Rats at P15–21 displayed seizure activity starting in the hippocampus and rapidly spreading to the neocortex after a short delay. SE was reached more rapidly in 15–21-day-old rats than in older animals. No morphological alterations have been reported in 3–10-day-old rats treated with pilocarpine up to 380 mg/kg. Only 5 of 14, 11–21-day-old rats that went through SE presented with some brain damage and the extent of this was less than that in adult rats. P24–60 rats presented with damage that was more similar to that observed in adult animals (Cavalheiro et al., 1987). In this study behavioural and EEG changes have been investigated only during the acute period. These data have been confirmed by Priel et al. (1996), who found that 7–17-day-old Wistar rats do not develop SRSs even when all of them received a high dose of pilocarpine (380 mg/kg). The latency to the appearance of the first seizure was longer in younger animals when compared to adult rats. The percentage of animals experiencing SRSs was higher when animals were injected at P50–120, and it decreased in younger rats. In addition, SRS frequency in the chronic phase was lower in the younger animal group (Priel et al., 1996). Hence, an age-dependent progression of seizure-related brain damage has been observed, with damage more severe in mature animals than in younger rats (Priel et al., 1996).

Epileptic activity, hippocampal damage and supragranular sprouting were not seen in P7–11 rats after a single dose of pilocarpine. However, three consecutive pilocarpine-induced SE episodes, during P7–9, induced an epileptic condition later in the life (Dos Santos et al., 2000; da Silva et al., 2005a). Single injections of pilocarpine for three consecutive days in P7–9 Wistar rats induced body tremor, scratching, forelimbs clonus and head bobbing that culminated in SE in all animals. Some experienced tonic–clonic convulsions that appeared to be more frequent after the second and third injection. Mortality with this approach was 0%. These animals displayed EEG alterations accompanied by motor seizures. Apoptotic neurons, corresponding to hippocampal interneurons, were identified in the dentate hilus (Dos Santos et al., 2000). EEG recordings made 1 month or more after the pilocarpine injections, have revealed abnormalities devoid of behavioural correlates. This activity was more intense and long-lasting in animals that presented with tonic convulsions during SE. However, the severity of the electrographic epileptiform features increased with age, being longer and more pronounced in older animals (Dos Santos et al., 2000). Interestingly, in the study by Dos Santos et al. (2000) animals exhibited hippocampal damage and developed complex partial seizures, but rats receiving the same treatment in another study (da Silva et al., 2005b) were found to undergo generalized electrographic seizures without marked hippocampal damage.

It seems likely that the occurrence of SE episodes during development induced complex cellular changes that can alter the maturation of neocortical and hippocampal circuits. In fact, developing rats, submitted to three consecutive episodes of pilocarpine-induced SE on P7, 8 and 9, presented with several long-lasting changes in neocortical architecture that could underlie the behavioural characteristics and the generalized epileptic discharges observed later in adulthood (da Silva et al., 2005b). The most prominent changes were altered intracortical microcircuitry development, decreased parvalbumin-positive cells in CA1 and dentate gyrus, increased glutamic acid decarboxylase 65 immunoreactivity in neocortex and altered neocortical apoptotic processes (da Silva et al., 2005b).

Administration of pilocarpine (30 mg/kg) to lithium-treated rats aged at P11–30, induced SE in all animals. Mortality was zero for animals between P11 and 14, and this increased to 33% and 50% in animals at P15–21 and P22–30, respectively. Higher doses of pilocarpine (60 mg/kg) evoked SE in all the animals, but with higher rates of mortality (67% at P11–14, reaching 100% at P15–21; Hirsch et al., 1992). Rats that experienced lithium–pilocarpine-induced SE at P10 did not develop SRSs and did not show any neuronal damage, but all adult rats undergoing lithium–pilocarpine-induced SE developed SRSs after a mean latent period of 24.9 days.

Significant neuronal loss was observed in hippocampus, basolateral and medial nuclei of amygdala, piriform cortex and lateral thalamic nucleus. Among rats exhibiting lithium-pilocarpine induced SE at P21, three groups of rats could be distinguished 2–3 months later (Dubé et al., 2001). The first group developed SRSs after a mean period of 74 days and the behavioural manifestations were very similar to those seen in adult rats. Rats in the second group did not develop SRSs but seizures could be triggered by stress. The third group developed neither SRSs nor stress-induced seizures. Significant neuronal damage was observed in the hilus of all three groups. Cell loss was found in the medial amygdala and layer II of the entorhinal cortex in the group without any seizures, and in the lateral thalamus and layer III–IV of the entorhinal cortex in rats that showed SRSs. Damage in layer II of the entorhinal cortex was seen in the triggered seizure group (Dubé et al., 2001). In the same study all adult rats injected with lithium–pilocarpine became epileptic and suffered massive and widespread cellular damage (Dubé et al., 2001).

In two reports originating from different laboratories (Thompson and Wasterlain, 1997; Towfighi et al., 2004), lithium–pilocarpine was tested in immature rabbits to assess any possible advantage compared to immature rats in seizure induction and sensitivity to neuronal damage. In the first study, P9 New Zealand White rabbit pups were treated with lithium (3 mEq/l, s.c.) and the following day with atropine methylbromide (1 mg/kg, s.c.) followed 15 min later by pilocarpine (200 mg/kg, i.p.). Approximately 80% of the animals suffered recurrent episodes of clonic seizures and postural alterations consisting of splaying of the limbs. The remaining animals exhibited a more severe response, with loss of posture and tonic–clonic seizures. In both cases, however, SE was not attained. Mortality was around 40%, and 65% of surviving rabbits presented neuronal loss in CA1 and, to a lesser extent, in CA3 and dentate hilus. Extra-hippocampal damage was also noted (Thompson and Wasterlain, 1997), thus these authors concluded that the immature rabbit was a better model to investigate seizure-induced damage than the neonatal rat.

In the other report, Towfighi et al. (2004) investigated the effects of pilocarpine treatment during the first and the second weeks postpartum, in the New Zealand white rabbits. The motivation to investigate rabbits originated from the fact this species, like humans, appears to be a “perinatal brain developer”. Groups of rabbits at P6–7 or P10–12, received 200–300 mg/kg of pilocarpine after pretreatment with lithium and atropine. Pilocarpine-induced seizures were scored according to a scale that identified motor behaviours such as chewing, tremors and extension of forelimbs as mild seizures. Recurrent clonic convulsions with splayed legs as well as robust tonic–clonic convulsions with apnea were scored as severe seizures. These phenomena persisted for less than 2 h and were accompanied by a moderate mortality (approximately 35%). In animals exhibiting severe seizures, brain damage was found in approximately 30% of animals lithium–pilocarpine-treated in postnatal week 1 and in around 65% of the animals treated in week 2. The most pronounced lesions were seen in CA1, followed by the subiculum, neocortex, CA3, basolateral and lateral amygdala and striatum (only in one animal). Towfighi et al. (2004) have also tested kainic acid as a convulsant, but were unable to induce seizures with this glutamatergic receptor agonist. Thus these studies again, showed an age-dependency of the damaging effects of pilocarpine-induced seizures.

## 10 Gender

Most laboratories have used male rats to limit the influences of variations in the activity of sex hormone-related axes and also because of the different modulatory effects of testosterone and estradiol on seizures (Galanopoulou et al., 2003). Studies aimed at comparing the different responses in Wistar males and females (Cavalheiro et al., 1987; Mello and Covolan, 1996; Ferreira et al., 2003) have failed to find evidence for any differences in behaviour, EEG or occurrence of SRSs. However, in another study Sprague–Dawley female rats were found to be less responsive to pilocarpine and to present lower mortality rates than male rats (Mejìas-Aponte et al., 2002). It is worth to note that the main aim of the study made by Mejìas-Aponte et al. (2002) was to compare susceptibility to seizures in males compared to female. In contrast, the other studies (Cavalheiro et al., 1987; Mello and Covolan, 1996; Ferreira et al., 2003) were not aimed at characterizing sex-dependent differences in the response to pilocarpine, thus the data obtained from females and males were pooled together. In addition, major sources of variability in these experiments were the lack of a precise timing of pilocarpine administration in relation to the pubertal development or, in mature female animals, in relation to the ovarian cycle. Fluctuations in female steroids are known to greatly affect the response to pilocarpine in terms both of survival and latencies to seizure onset (Valente et al., 2002).

Findings obtained from experiments performed on female rats in relationship to physiological changes in the sex hormonal axes, indicate that seizure frequency decreases during pregnancy and lactation. Significant changes in gonadal, hypophyseal and hypothalamic hormones, as well as reduction in fertility, increase in mating time and decrease of the gestational period occurred in female Wistar pilocarpine-treated rats (Amado and Cavalheiro, 1998). In addition, it has been reported that castration interfered with epileptogenesis in the pilocarpine model of epilepsy (Valente et al., 2002). In fact these authors observed an increased mortality rate during SE, shorter latent period, and more pronounced hippocampal cell loss in castrated animals. These data suggest that female sexual hormones may have protective effects against pilocarpine-induced SE. In line with this view, it has been reported that ovariectomized females present with increase mortality rate, shorter time to onset of SE and more pronounced mossy fibre sprouting (Valente et al., 2002).

## 11 Discussion

### 11.1 Homology with TLE aetiology

TLE is a major neurological disease which presents a poor response to AEDs. In a recent study (Mathern et al., 2002), 54% of TLE patients undergoing surgical treatment for pharmacoresistance were found to present hippocampal sclerosis only, and 17% presented hippocampal sclerosis associated with another lesion. In this investigation, 71% of patients with hippocampal sclerosis suffered of seizures in the childhood or, less frequently, later. According to Bruton (1988), hippocampal sclerosis is associated with childhood febrile convulsions in 55% of TLE patients, birth injury or cerebral trauma in 24%, and with SE in 18%. In few cases it was not possible to find any “initial precipitating injury” that could be interpreted as a possible cause of hippocampal sclerosis (Mathern et al., 2002).

Among the several types of initial precipitating injuries, those mimicking SE are more feasibly applied in animals in view of the very simple approach offered by the i.p. injection of a convulsive drug such as pilocarpine. Thus, these models do not require any apparatus or particular technical ability, as instead in the case of cerebral trauma or febrile seizures. For the same reason, they do not have additional costs apart from drugs and animals. These characteristics have made the pilocarpine and kainic acid models particularly appealing. Are these reasons sufficient for considering these models better than others in mimicking TLE? We believe this is the case. However, we have also to consider whether SE or prolonged seizures are a frequent clinical condition that could thus be proficiently modeled to study TLE.

When considering the original definition of initial precipitating injury, indicating any event causing loss of consciousness for more than 30 min or the alteration of cognition for more than 4 h, it turned out that more than 40% of injuries were caused by prolonged seizures or SE (Mathern et al., 1997a). Indeed, we have shown that just 30-min SE (the minimal time interval generally accepted to define SE) are enough to reproduce the pilocarpine model (Biagini et al., 2008). Thus, by reducing the period of seizure duration may result in a better adherence of the pilocarpine model to the clinical history of TLE patients.

Another critical point is the age dependency of TLE. Initial precipitating injuries occur more frequently in the first year of age or, anyway, in the childhood (Mathern et al., 2002). Convulsive SEs, which occurs in 51/100,000/year children under 1 year of age, are less frequent in children between 1 and 4 years of age (29/100,000/year) and drops abruptly over 5 years of age (Raspall-Chaure et al., 2007). As we described in Section 9, it is difficult to reproduce all the features of the pilocarpine model until 21 days of age. Undoubtedly, this is a drawback of the model that depends on the various differences in central nervous system development between rodents and humans. Focusing on 3-week-old rats could provide an improvement in the pilocarpine model homology, but certainly it will not satisfy the requirement for an ideal model. In fact, working on young animals could be more expensive and time consuming than with adult animals, as the young animals present higher mortality rates and a longer latent period. However, there is no doubt that the longer latency to SRSs in young animals offers a better homology with the human disease.

### 11.2 Homology with TLE pathophysiology

Many pathophysiological mechanisms associated with epileptogenesis in the pilocarpine model have been demonstrated to occur also in the human hippocampal formation, including mossy fibre sprouting, interneuron loss and granule cell dispersion in the dentate gyrus (Mathern et al., 1997a; Wieser, 2004). These aspects have been already considered in previous review articles (Covolan and Mello, 2000; Avoli et al., 2002; Pitkänen and Sutula, 2002). However, the discussion has generally been focused on the changes occurring in the hippocampal formation, whereas extrahippocampal lesions have been systematically underevaluated.

In the pilocarpine model, damage is widespread and involves several brain regions (as illustrated in Sections 2, 3 and 5). The presence of multiple lesion sites has been considered as a drawback of the model (Sloviter, 2005), maybe because the attention has been focused mainly on hippocampal sclerosis, the hallmark of TLE. However, neuronal lesions have been described also in the amygdala, thalamus and neocortex of TLE patients (Mathern et al., 1997a). Whatever lesion is found, the main question that remains is in which way SRSs are generated in TLE and in the respective animal model. To this regard, Zhang et al. (2002) have shown that with repeated subthreshold kainic acid-pilocarpine doses, rats finally develop SE and become epileptic without evident neuronal loss in the major cerebral areas in which damage usually occurs. Thus, epileptogenesis apparently can take place without any gross lesion to specific brain regions.

These data also indicate that the problem is not to set the ideal model of TLE pathophysiology but to correctly interpreter what the animal model used is suggesting us that could be relevant for understanding the mechanisms involved in this disease. Thus, the various variants of the pilocarpine model could be interesting to analyse different aspects of TLE. For instance, the model based on repeated subthreshold kainic acid-pilocarpine injections proposed by Zhang et al. (2002) is certainly interesting to study the cellular and molecular mechanisms involved in epileptogenesis, but not those that are implicated in neurodegeneration. In contrast, the standard pilocarpine paradigm is suited to address how damage develops in selected brain regions during seizures. To this regard, it has to be noticed that TLE may be considered a degenerative disease as shown by studies that have proven a progression in hippocampal and extrahippocampal atrophy in relation to the duration of refractory seizures (Fuerst et al., 2003; Bernasconi et al., 2005; Bonilha et al., 2006). Interestingly, it has been recently shown the presence of multiple foci of blood brain barrier damage in the brain of animals presenting SRSs and in TLE patients refractory to AEDs (van Vliet et al., 2007).

### 11.3 Homology with TLE drug treatment

It is well known that hippocampal sclerosis is associated with a poor response to AEDs in as much as 75% of patients (Spencer, 2002). Thus, an absolute requirement for the pilocarpine model is the homology with pharmacoresistance. Only a few studies on pharmacoresistance have been however conducted in pilocarpine-treated rats. Probably, the reason for such scarce investigation on this issue is that pilocarpine-treated epileptic rats are rather difficult to be managed because they are very aggressive; in addition, pilocarpine-treated epileptic rats are also very sensitive to stress and require a way of drug administration that might prevent the possibility to elicit seizures.

In the few studies published to date, AEDs were administered by i.p. injection (Leite and Cavalheiro, 1995; Chakir et al., 2006) or s.c. via osmotic minipumps (Glien et al., 2002). Leite and Cavalheiro (1995) were the first to administer AEDs to pilocarpine-treated animals and basically invalidated the usefulness of this model in mimicking TLE pharmacoresistant patients. As already mentioned, other two independent studies observed a poor response of lithium–pilocarpine-treated female rats to levetiracetam (Glien et al., 2002) and of pilocarpine-treated male rats to carbamazepine (Chakir et al., 2006), suggesting that the model could be able to mimic also pharmacoresistance. There is no simple explanation for these discrepancies, but we have obtained preliminary results that suggest that the response of pilocarpine-treated epileptic rats to AED treatment is greatly influenced by the degree of hippocampal damage (Longo, 2005). This hypothesis is reinforced by data obtained in a different model of pharmacoresistance in which rats non responding to phenobarbital presented with neuronal cell damage in the hippocampus, while those responding to this barbiturate had a structurally preserved hippocampus (Volk et al., 2006).

## 12 Conclusions

Based on the evidence collected from different laboratories, it appears that the pilocarpine model may represent a suitable tool for investigating the pathophysiogenetic mechanisms of TLE. The initial insult – which may be analogous to febrile seizures, perinatal hypoxia, infections or head trauma in humans and it is represented by SE in the pilocarpine model – is followed by the development of chronic seizures after a latent period of variable duration. It has been demonstrated that pilocarpine-induced SE, when not prematurely interrupted by drug administration, is able to induce anatomical damage and reorganization similar to that observed in humans affected by TLE. However, since it has been shown that age, strain, gender, as well as combination with other drugs can be critical factors for the development of chronic epilepsy, all these variables have to be carefully controlled to obtain a reliable animal model of TLE, as well as for reducing mortality and variability as far as possible while recreating the full epileptic disorder.

It has been underscored that the inconsistencies found in the way to implement the pilocarpine model in various laboratories could lead to conclusions that are irrelevant or even misleading for TLE. In addition, there is danger of ignoring findings that could be of help in understanding some aspects of TLE pathophysiology. Indeed, the first requirement for the pilocarpine model is face validity with TLE, so the presence of an initial precipitating injury, a latent period, seizures origin in the temporal lobe and associated structures and, possibly but not necessarily, the presence of hippocampal lesions with a topography similar to what found in the human. The presence of lesions extending in areas not directly connected with the hippocampus somehow limits the value of this model, especially when substantia nigra is involved.

We propose that different laboratories should provide a common effort to limit SE duration (30–60 min appears to be enough) to confine neuronal damage to the hippocampal formation and related limbic regions. As already mentioned, the hippocampus proper, the entorhinal cortex and the amygdala appear to be the most damaged areas in patients affected by TLE. In this regard, it may also be important to unify the drug treatment that is employed to terminate SE. As diazepam appears to be the most widely (although not the most effective) drug used, it should be administered at the same dose in the various laboratories, possibly at 20 mg/kg injected i.p. in a single administration. Indeed, we have recently obtained evidence that limiting SE to 30 min by quelling seizures with diazepam 20 mg/kg results in epileptic rats with lesions well confined in the limbic system (Biagini et al., 2008). We also recommend employing adult 7–8-week-old Sprague–Dawley rats since these animals are characterized by a higher survival rate when using a 30-min SE.

Finally, another point is the predictive validity of this model, i.e. the ability to reproduce the same pattern of responsiveness to AEDs seen in TLE patients. This point has already been reviewed extensively (Turski et al., 1989; Loscher, 2002). As discussed in Section 11, the ability to reproduce pharmacoresistance (i.e., to what extent pilocarpine-treated rats develop seizures refractory to AEDs) remains controversial. According to Glien et al. (2002), levetiracetam can decrease but not abolish seizures in the lithium–pilocarpine model. Interestingly, in this experiment levetiracetam was administered via miniosmotic pumps thus ruling out the possible interference of stress in inducing seizures. This is an innovative approach to drug administration in epileptic rats that should be preferred to that based on the i.p. injection of multiple antiepileptic doses (up to 3/day, Leite and Cavalheiro, 1995; Chakir et al., 2006), obviously causing more distress (Doheny et al., 2002). Thus, it could be possible to demonstrate the homology of this model with the human disease by showing that different populations of rats are responsive or not, respectively, to AED treatment. In this eventuality, the pilocarpine model could serve as a powerful tool to predict the response of TLE patients to new antiepileptic treatments. However, further studies are required to definitively clarify this point since different populations of drug-responsive or drug-resistant rats still have not been found in the pilocarpine model.

In conclusion, the pilocarpine model of TLE remains an animal model relevant for the human disease after 25 years from its initial characterization. Although it has been used by several laboratories in order to investigate the consequences of initial seizures as well as the processes leading to establish the chronic disease, it has still to be thoroughly investigated in some aspects intrinsic to the model, including the relations among several parameters such as SE intensity, neuronal damage, latent period length and sensitivity to AEDs. We predict that the contribution of investigators to clarify these aspects in the pilocarpine model will allow a more rational approach to understanding the multifaceted aspects of TLE.

## Figures and Tables

**Table 1:** Mortality rates found in different laboratories by investigating the pilocarpine or the lithium–pilocarpine models

Pilocarpine dose (mg/kg)	SE duration (min)	Mortality (%)	Mortality mean ± S.D.	References
320–360	90	17^b^		Williams et al. (2002)
	120	55^a^	37.3 ± 19.1	Esclapez et al. (1999)
	>120	40^a^		Goffin et al. (2007)

380	>120	5^b^		Poirier et al. (2000)
	>120	30^a^	22.0 ± 14.7	Leite et al. (1990)
	>120	31^b^		Liu et al. (1994b)

30 (lithium pretreatment, 3 mEq/kg)	90	45^a^		Fujikawa et al. (1999)
	>120	24^b^	56.0 ± 39.2	Glien et al. (2001)
	>120	100^a^		Fujikawa et al. (1999)

Note the wide variability found in the different experimental sessions even when using similar experimental protocols. These discrepancies suggest that environmental variables difficult to be controlled could influence the animal response to SE induction, resulting in a different survival probability. The rat strain also appears to be important, as the lowest mortality rates have been observed in Sprague–Dawley rats.

a Wistar rats (mortality rate: 54.00 ± 27.25).

b Sprague–Dawley rats (mortality rate: 19.25 ± 11.09).

**Table 2:** Differences in the response to pilocarpine in various mouse strains

Strain	Tonic-clonic seizure stage 5 (%)	Mortality (%)	Neuronal damage (CA3–CA1)	Mossy fibre sprouting	References
C57BL/6	100	21	++	++	Shibley and Smith (2002)
	42.9	19	++	++	Chen et al. (2005)

CD1	61.5	21	++	++	Shibley and Smith (2002)
	23.5	29.4	++	++	Chen et al. (2005)

FVB/N	55.6	60.3	+	++	Chen et al. (2005)
A/J	100	75	+++	+++	Winawer et al. (2007)
DBA	90	17	+++	++	Winawer et al. (2007)

In agreement with findings in rats, the mouse strain greatly affects the response in terms of mortality rate, that was found to be higher in FVB/N and A/J inbred mice. The probability to get fully developed seizures is also greatly affected by the mouse strain but, in this case, variability is very large. Neuronal loss is, in general, less pronounced in mice than in rats with the exception of A/J and DBA inbred strains. Note that CD1 is the only outbred strain characterized in these studies. + mild; ++ moderate; +++ severe.
